# Treatment Advances in EBV Related Lymphoproliferative Diseases

**DOI:** 10.3389/fonc.2022.838817

**Published:** 2022-04-19

**Authors:** Kebing Lv, Ting Yin, Min Yu, Zhiwei Chen, Yulan Zhou, Fei Li

**Affiliations:** ^1^ Center of Hematology, The First Affiliated Hospital of Nanchang University, Nanchang, China; ^2^ Institute of Hematology, Academy of Clinical Medicine of Jiangxi Province, Nanchang, China; ^3^ Clinical Research Center for Hematologic Disease of Jiangxi Province, Nanchang, China; ^4^ Institute of Lymphoma and Myeloma, Nanchang University, Nanchang, China

**Keywords:** barr virus, lymphoproliferative disorders, lymphoma, therapy, advances

## Abstract

Epstein Barr virus (EBV) can affect 90% of the human population. It can invade B lymphocytes, T lymphocytes and natural killer cells of the host and remain in the host for life. The long latency and reactivation of EBV can cause malignant transformation, leading to various lymphoproliferative diseases (LPDs), including EBV-related B-cell lymphoproliferative diseases (EBV-B-LPDs) (for example, Burkitt lymphoma (BL), classic Hodgkin’s lymphoma (cHL), and posttransplantation and HIV-related lymphoproliferative diseases) and EBV-related T-cell lymphoproliferative diseases (EBV-T/NK-LPDs) (for example, extranodal nasal type natural killer/T-cell lymphoma (ENKTCL), aggressive NK cell leukaemia (ANKL), and peripheral T-cell lymphoma, not otherwise specified (PTCL-NOS). EBV-LPDs are heterogeneous with different clinical features and prognoses. The treatment of EBV-LPDs is usually similar to that of EBV-negative lymphoma with the same histology and can include chemotherapy, radiotherapy, and hematopoietic stem cell transplant (HSCT). However, problems such as serious toxicity and drug resistance worsen the survival prognosis of patients. EBV expresses a variety of viral and lytic proteins that regulate cell cycle and death processes and promote the survival of tumour cells. Based on these characteristics, a series of treatment strategies for EBV in related malignant tumours have been developed, such as monoclonal antibodies, immune checkpoint inhibitors, cytotoxic T lymphocytes (CTLs) and epigenetic therapy. These new individualized therapies can produce highly specific killing effects on tumour cells, and nontumour cells can be protected from toxicity. This paper will focus on the latest progress in the treatment of EBV-LPDs based on pathological mechanisms.

## Background

**Figure 1:**
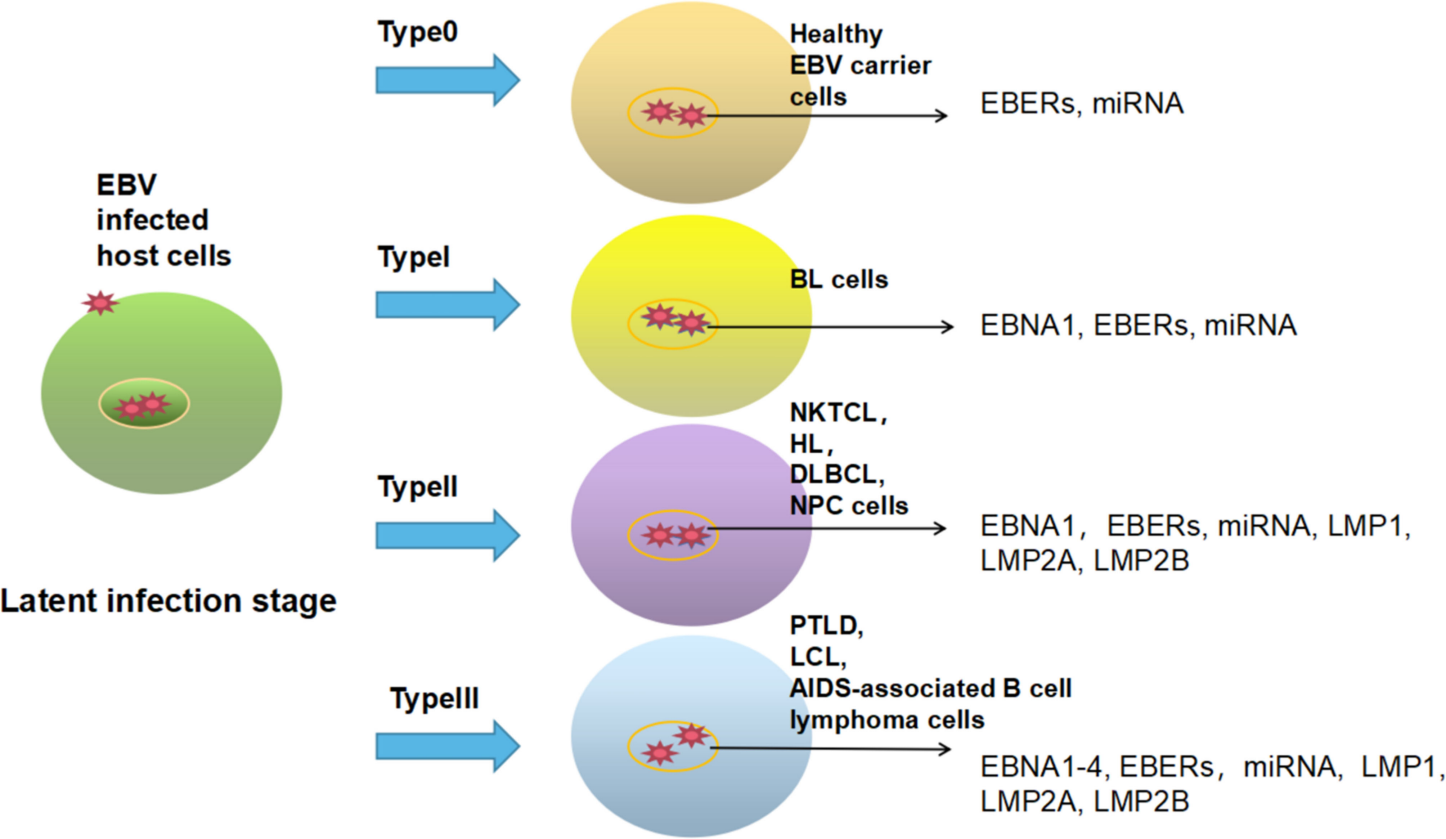
EBV expresses different viral proteins in different host cells during a latent infection. Latent type 0: EBER and miRNA are expressed in the nucleus of host cells, mainly in EBV carriers in good health. Latent type I: EBNA1, EBER and miRNA are commonly expressed in patients with Burkitt lymphoma (BL) (5). Latent type II: EBNA1, EBER, miRNA, LMP1, LMP2A, and LMP2B are generally expressed in patients with HL, nasal NK/T cell lymphoma (NKTCL), NPC and diffuse large B-cell lymphoma (DLBCL) (6, 7). Latent type III: EBNA1–4, EBER, miRNA, LMP1, LMP2A and LMP2B can be expressed in posttransplant lymphoproliferative disease (PTLD), lymphoblastic cell line (LCL) and AIDS-associated B-cell lymphoma (8).

**Figure 2:**
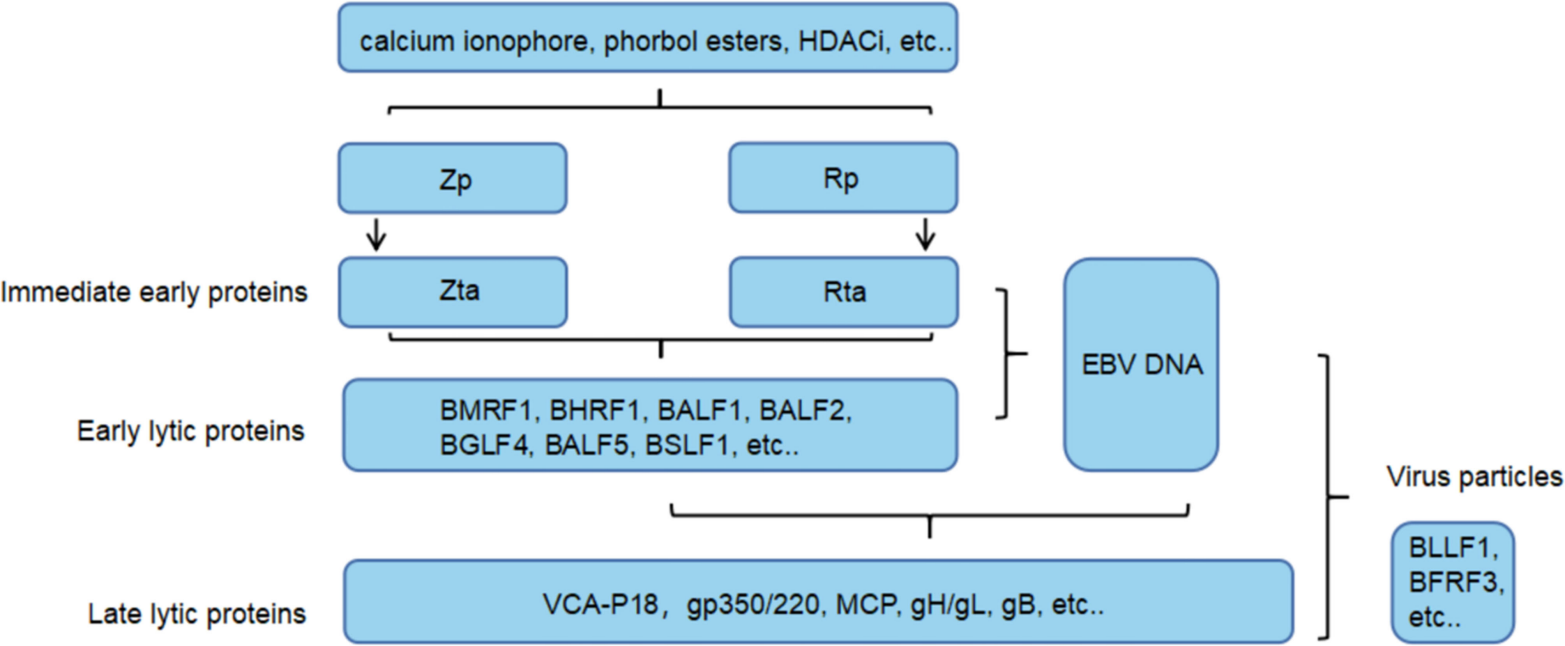
EBV lytic cycle reactivation. After a variety of stimulants activate the EBV Z/R promoters (Zp and Rp), the two immediate early proteins reciprocally activate expression and drive the EBV lytic cycle. In this process, a series of lytic proteins are produced to regulate gene expression and induce DNA replication. Finally, the virus particles are packaged by structural proteins and released through exocytosis.

Epstein Barr virus (EBV) is classified as a gamma-1 herpesvirus, and people are generally susceptible to this virus. Patients with EBV infection are usually asymptomatic, and the development of symptomatic disease is associated with delayed primary infection, leading to infectious mononucleosis in young people. In addition, EBV infection is associated with various EBV-related malignancies, such as nasopharyngeal carcinoma (NPC), gastric cancer subtypes, and several lymphoproliferative diseases (LPDs), especially B-cell and T-cell lymphomas (1, 2). It causes about 200000 new cancer cases worldwide every year. In cancer cells, EBV usually remains latent to escape human immune surveillance but can switch from a latent to a lytic cycle to infect new cells in response to physiological stimuli (3, 4). There are three groups of viral proteins expressed by EBV during a latent infection: (1) the Epstein Barr virus nuclear antigen (EBNA) family, which includes EBNA1–EBNA4, within which EBNA3 encompasses EBNA3a, EBNA3b and EBNA3c; (2) the late membrane protein (LMP) group, which includes LMP1 and LMP2; and (3) EBV-encoded RNA (EBER). These viral proteins follow four latent expression patterns, and different host cells express different viral proteins (3) (**Figure 1**). In cells infected with latent EBV, the immediate early (IE) proteins BZLF1 (Zta) and BRLF1 (Rta) are key for mediating transformation, but the two promoters that control Zta and Rta gene transcription are inactive (4). Upon induction by stimulants that activate B cells, such as calcium ionophores, phorbol esters and histone deacetylase (HDAC) inhibitors, these promoters are activated, resulting in the expression of immediate early lytic proteins, followed by the production of early lytic proteins (BMRF1, BALF1, BHRF1, BSLF1, etc.) and the initiation of viral DNA replication (9, 10). At the same time, early lytic proteins and DNA replication trigger the expression of late lytic proteins (VCA-p18, gp350/220, MCP, gH/gL, etc.), which eventually leads to the production of infectious virus particles such as BLLF1 and BFRF3 (4, 9). More than 70 viral proteins can be produced in the entire process (11) (**Figure 2**).

There is increasing evidence that virus latent and lytic proteins can maintain the proliferation and survival of EBV-positive cancer cells by influencing cellular mechanisms regulating the cell cycle, apoptosis and immune recognition of host cells. Research to identify therapeutics for EBV has produced many surprising results. The specific killing effect not only improves the curative effect but also greatly reduces toxicity and side effects. Therefore, in addition to chemotherapy, regulatory immunosuppressive therapy and allogeneic hematopoietic stem cell transplant (allo-HSCT), different EBV-based treatment strategies, such as immunotherapy, gene therapy and epigenetic therapy, are research hotspots. This paper will summarize EBV-LPD treatment methods and discuss research advances regarding these new schemes.

## EBV-Associated B-cell Lymphoproliferative Disorders

### Chemotherapy

EBV was initially detected by Epstein MA et al. in isolated and cultured cells from a BL child in Uganda (12). BL is a highly proliferative B-cell non-Hodgkin lymphoma (NHL) that can be divided into three different variants: endemic (African) BL, sporadic BL and immunodeficiency-related BL (13). EBV can be detected in almost all cases of endemic BL and up to 40% of cases of immunodeficiency-related BL (14). Various intensive treatment schemes have shown great activity in patients with BL. Previous research found that CODOX-M/IVAC +/- R (cyclophosphamide, vincristine, doxorubicin, methotrexate, ifosfamide, etoposide, and cytarabine with or without rituximab), CALGB (Cancer and Leukemia Group B), Hyper-CVAD +/- R (cyclophosphamide, vincristine, doxorubicin, and dexamethasone with or without rituximab), and dose-adjusted (DA) R-EPOCH (rituximab, etoposide, prednisone, vincristine, cyclophosphamide, and doxorubicin) achieved significant results. The best results showed that patients can achieve 95% event-free survival (EFS) and 100% overall survival (OS) (15–20) (**Table 1**). The low-dose adjusted R-EPOCH regimen represents major progress, allowing the treatment of elderly and human immunodeficiency virus (HIV)-positive patients and solving the problem of limited application of a high-intensity regimen due to severe toxicity in these patient groups. However, for patients with endemic diseases, treatment schemes are usually limited by cost, and different low-income and middle-income countries approve different schemes. For example, the OS rate of patients in sub-Saharan Africa is between 51% and 67%, which is much worse than that in resource rich countries (38). Therefore, those successful studies cannot represent the heterogeneous population. Meanwhile, chemotherapy is not sufficient to block disease activity and eradicate infected cells. There is a lack of further salvage treatment for patients with recurrent or refractory BL, and there is an urgent need to develop more effective treatments.

**Table 1:** Summary of available therapies in EBV-B-LPD.

Therapy			Patients characteristics	Efficacy	Reference
Chemotherapy		CODOX-M/IVAC+/-R	BL	2-year EFS: 65%, 2-year OS: 73%; 3-year PFS: 74%, 3-year OS: 77%	(15, 16)
		GALGB+/-R	BL	3-year EFS: 74%, 2-year OS: 78%; 3-year EFS、OS: 45-54%	(18, 19)
		HyperCVAD +/-R	BL	3-year EFS: 80%, 3-year OS: 89%	(17)
		(DA)R-EPOCH	BL	EFS: 95%, OS: 100%	(20)
Immunotherapy	Monoclonal antibody	R-CHOP	PTLD/DLBCL	First-line treatment	–
		Rituximab	PTLD	It is related to the elimination of PTLD related mortality	(21)
		BV	PTLD/DLBCL	The patient had no disease progression 3.5 years after PTLD.	(22)
		Daradaratumumab	PTLD	A rapid decrease in EBV viral load was observed	(23)
	Checkpoint inhibitors	Nivolumab	HL	ORR: 66%	(24)
			HL	ORR: 89%, CR:59%	(25)
		Pembrolizumab	HL	ORR: 69%, CR: 22.4%	(26)
		Sintilimab	HL	ORR: 80%	(27)
		Tislelizumab	HL	CR: 62.9%, ORR: 87.1%	(28)
		Camrelizumab	HL	CR: 28%, ORR:76%	(29)
		Nivolumab + BV	HL	CR: 67%, ORR:85%	(30)
			HL	OS: 98%	(31)
			HL	It is effective and well tolerated in the elderly	(32)
		Nivolumab	PTLD	The child received CR	(33)
		Nivolumab		The woman received CR	(34)
	CTL	EBV-CTL	PTLD	CR: 84.6%	(35)
		EBV-CTL line bank	PTLD	The 6 months effective rate was 52%	(36)
		CMD-003	R/R lymphoma and PTLD	It has been granted fast track by FDA	(3)
		EBV-CTL and LMP-2-CTL	cHL	The patients were well tolerated and sustained clinical responses were observed.	(37)

### Immunotherapy

#### Monoclonal antibodies

EBV is detected in 25–50% of Hodgkin’s lymphoma (HL) cases in the United States and Europe (39, 40). In immunosuppressed individuals, EBV-encoded LMP-1 contributes to NF-κB pathway activation and induces an antiapoptotic phenotype in Reed–Sternberg (RS) cells in HL (41). CD30, a transmembrane protein belonging to the tumour necrosis factor receptor family, is specifically expressed in normally activated (rather than static) B and T cells and NK cells. The CD30-targeted antibody brentuximab vedotin (BV) showed significant antitumour activity in recurrent or refractory (R/R) HL and anaplastic large cell lymphoma (ALCL), leading to its accelerated approval by the FDA (42). The results of a preliminary phase II study showed moderate efficacy of SGN-30 in HL patients with all levels of CD30 expression (43). In addition, continuous treatment with MDX-060, a human anti-CD30 immunoglobulin G1 monoclonal antibody, was well tolerated in phase I and II clinical trials. However, there has been no study on the effect of CD30 mAbs on EBV load.

In 2003, EBV+ diffuse large B cell lymphoma (DLBCL) was first described as a unique entity in elderly patients, and *in situ* hybridization showed that this disease was related to EBV (44). In 2008, the World Health Organization (WHO) defined the temporary entity of DLBCL based on a large number of studies conducted in Asian populations and named it ‘‘EBV positive DLBCL of the elderly”. After that, several groups studied the association between EBV and DLBCL in children and young people, and proved that EBV positive was also detected in these age groups (45–50), and some of which showing a larger morphological spectrum and better survival rate (51). Therefore, the term “elderly” was replaced with “not otherwise specified” (EBV + DLBCL, NOS) in the 2016 classification and is no longer considered provisional (52). Sarah Park et al. evaluated EBER expression by *in situ* hybridization in 380 samples from DLBCL patients to evaluate the significance of EBV positivity on the survival and prognosis of DLBCL patients. These researchers found that patients with EBER-positive DLBCL showed more rapid clinical deterioration and worse survival rates and treatment responses (53). CD20 is a nonglycosylated pan B-cell transmembrane phosphoprotein with a molecular weight of 35 KD that is expressed on the surface of most mature B cells. At present, rituximab, which targets CD20, combined with chemotherapy is the first-line treatment for EBV+ DLBCL. Different reactions to R-CHOP (rituximab, etoposide, prednisone, vincristine, cyclophosphamide, and doxorubicin) have been reported worldwide, but no prospective comparative study has been conducted (54). CD30 is expressed in some posttransplant lymphoproliferative diseases (PTLDs) induced by EBV infection, including DLBCL. Brentuximab vedotin (BV), a CD30-directed antibody–toxin conjugate, represents an attractive treatment. Thomas Mika et al. reported the first case of long-term control/cure of highly invasive EBV-DLBCL by combining BV and adoptive EBV-specific T cell therapy. The patient had no disease progression 3.5 years after PTLD. However, the long-term efficacy of BV monotherapy in the treatment of CD30+ DLBCL caused by PTLD has not been determined (22). Clinical trials of BV combined with other therapies are ongoing (**Table 3**). Other therapies were mainly studied in the background of PTLD, which has extensive effects on EBV+ DLBCL.

Reactivation of EBV after bone marrow transplantation usually leads to a LPD that does not respond well to standard treatment and is usually fatal. As early as 1969, the incidence rate of LPDs in solid organ and bone marrow transplant recipients was very high, between 0.5% and 17% (79, 80). EBV infection affects approximately 60–80% of patients with PTLD, including 100% of patients with early-onset PTLD (81). Associated EBV infection and CD20+ B cell proliferation were observed in 90% of cases. Therefore, in addition to stopping immunosuppressive drugs, monoclonal anti-CD20 antibodies (such as rituximab) can be used in standard treatment (82, 83). W J F M van der Velden et al. noted that pre-emptive treatment, which is defined as rituximab administration to patients with a symptomatic EBV infection, is related to the elimination of PTLD-related mortality (21). At present, there is no standardized rescue treatment. If there is no response to anti-CD20 treatment, patient prognosis is very poor. Patrick-Pascal Strunz et al. reported the first case of combined treatment with a CD38 antibody (daratumumab) and EBV-specific cytotoxic T lymphocytes for EBV+ rituximab-refractory PTLD. The flow cytometry results of a sample from the 55-year-old male patient showed the loss of CD20 and continuously high CD38 expression in the homogeneous B cell population. Therefore, after the administration of EBV-specific cytotoxic T lymphocytes to this patient, daratumumab was added, and a rapid decrease in EBV viral load was observed. However, the examination results showed early recurrence of PTLD after 2 weeks (23). The role of CD38-targeted immunotherapy in the treatment of rituximab-refractory CD38+ PTLD needs to be further explored. In addition, the CD30 antibody mentioned above is also used to treat PTLD.

#### Checkpoint Inhibitors

**Figure 3:**
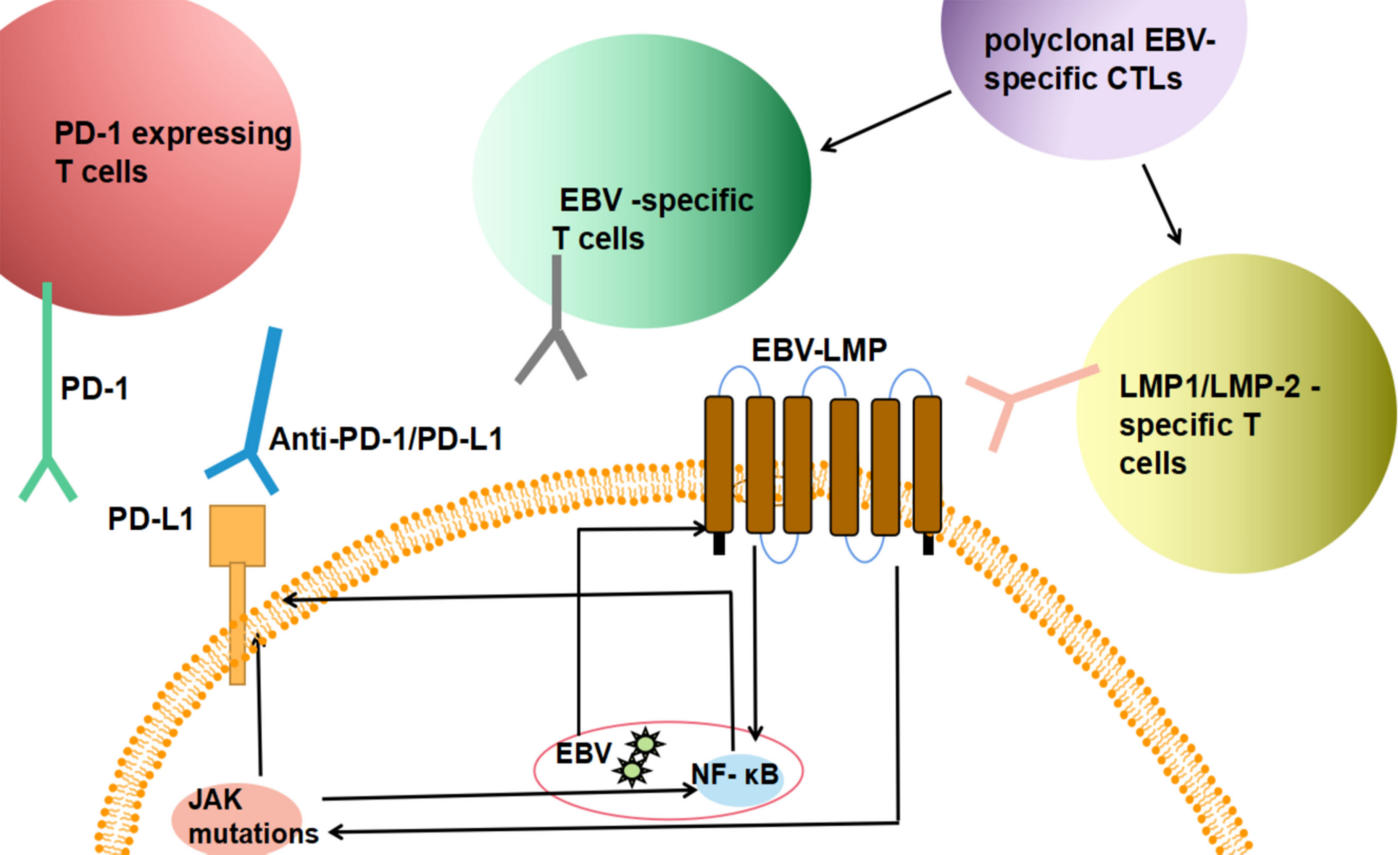
Summary of the immunotherapeutic mechanism of EBV infected tumor cells. EBV infected tumor cells express PD-L1, and binding with PD-1-expressing T cells in microenvironment can inhibit immune killing function; EBV drives the overexpression of latent membrane protein LMP and the activates NF- κB/MAPK and JAK/STAT signaling pathways lead to high expression of PD-L1. Therefore, after the infusion of polyclonal EBV specific CTLs, the increased frequency of EBV and LMP specific T cells and the application of PD-1/PD-1/L1 drugs can reduce immunosuppression and kill tumour cells.

Programmed death ligand 1 (PD-L1) is an immunomodulatory molecule expressed by antigen-presenting cells that selectively binds to the PD-1 receptor on T cells to inhibit T cell immune function. Both RS cells of cHL and malignant B cells of PTLD express PD-L1, and this expression is promoted by EBV. Therefore, these diseases share this immune escape mechanism (35, 36, 84) (**Figure 3**). Nivolumab and pembrolizumab, humanized anti-PD-1 monoclonal antibodies, have been proven to be effective in the treatment of patients with R/R cHL. In 2016, Anas Younes and his team found that the overall response rate (ORR) for nivolumab was 66% with acceptable safety in cHL patients who failed autologous stem cell transplant (ASCT) (24). In 2017, a phase II study involving 221 R/R HL patients showed that the ORR for pembrolizumab was 69%, and the complete remission (CR) rate was 22.4% (26). Subsequent extended follow-up reported that the median PFS was 14 months (85). Nivolumab and pembrolizumab were approved by the FDA in 2016 and began to be used in the treatment of cHL in R/R patients in 2017. In addition, pembrolizumab can be included in consolidation treatment after ASCT. Philippe Armand et al. conducted the first immune checkpoint blocking study for consolidation therapy in patients with R/R cHL and proved that PD-1 blockade with pembrolizumab after ASCT had acceptable safety and a significant progression-free survival (PFS) benefit (86). A large number of combined therapy studies including PD-1 have emerged (**Table 1**). The combined use of BV-nivolumab is a promising scheme. In a single arm phase I/II study, BV-nivolumab was applied to patients before ASCT, achieving a high CR of 67% and an ORR of 85% (30, 87). Among patients with high-risk R/R HL, consolidation after HCT with BV-nivolumab resulted in an estimated 18 month PFS and OS of 95% and 98% in all 59 patients, respectively (31). In addition, the combination of the two drugs is effective and well tolerated in newly treated elderly patients with HL (32). There are also a variety of new PD-1 inhibitors, such as sintilimab, tirizumab and camerezumab, which are under development. Their ORR is about 80% and CR rate is 30 – 60% (27–29) (**Table 1**). These studies excluded patients previously treated with PD-1. In the future, it will be interesting to assess the response of patients previously exposed to checkpoint inhibitors to new PD-1 inhibitors. Given the known efficacy in cHL, the use of immune checkpoint inhibitors in PTLD is promising (88). It is reported that after all conventional treatments failed and significant toxicity occurred in a child with PTLD, the salvage treatment with nivolumab achieved CR (33). Similarly, this result was also observed after application of nivolumab in a woman with cHL-like PTLD (34). At present, a phase I clinical trial to explore the efficacy of nivolumab and EBV-specific T cells in patients with R/R EBV-positive lymphoma, including PTLD, is completed, but no results were released (NCT02973113).

#### CTLs

Immunotherapy strategies to restore virus-specific immunity are an attractive alternative to antiviral therapy. EBV-specific CTLs of human leukocyte antigens (HLA)-matched donors or autologous lymphocytes can be activated and expanded *in vitro* and then injected into the recipient, where they can restore cellular immunity after EBV infection and eradicate EBV-infected cells. The most common adverse reaction is graft-versus-host disease (GVHD). Compared with adoptive therapy with monoclonal antibodies, CTLs can actively migrate through the microvascular wall to reach isolated tumour cells and undergo self-expansion. CTLs can kill tumour cells through cytotoxic effectors and have advantages in biological distribution and antitumour activity (89). In some initial single-centre experiments, EBV-CTLs were used to treat viral reactivation after bone marrow transplantation and achieved clear results (90, 91). In 1994, Ekaterina Doublovina et al. reported that five patients with EBV-associated lymphoma achieved sustained clinical remission after receiving peripheral blood mononuclear cells (PBMCs) from EBV-seropositive transplant donors (92). Subsequently, C M Rooney et al. prepared the first EBV CTL line *in vitro* from donor leukocytes and infused these cells into ten lymphoma patients after allogeneic transplantation to reconstruct EBV-specific immunity; the cells were found to persist for ten weeks *in vivo* without GVHD (93). The team infused EBV-CTLs in 114 HSCT patients at three different centres over a 12-year period. The CR rate reached 84.6% in 13 patients with PTLD and proved that the duration of functional CTLs was up to 9 years. The other patients who received preventive treatment did not develop PTLD [42]. The main disadvantages of generating EBV-CTLs for specific patients are that it is expensive and time-consuming; it takes three to four months to generate a suitable CTL system. Tanzina Haque et al. generated allogeneic virus-specific CTL line banks from normal donors, overcoming the main limitations. It was found that the higher the HLA match between patients and CTLs was, the better the response. In this phase II clinical trial, the 6-month response rate of 33 patients with PTLD who failed conventional treatment was 52% (94). In addition, the autologous T cell therapy CMD-003 has been approved by the FDA for the treatment of R/R lymphoma and PTLD (3) (**Table 1**). Due to cost constraints and technical difficulties, EBV-specific CTLs are always used after the patient’s condition has deteriorated, and this treatment strategy is not currently being used in the clinic. A multicentre, open label, single arm, phase III study was completed in March 2021. In the context of allogeneic haematopoietic cell transplantation, the efficacy and safety of an allogeneic T cell immunotherapy called tabelecleucel in the treatment of EBV+ PTLD were evaluated. The results have not been published (NCT03392142). Other relevant clinical trials are still in progress (**Table 3**).

RS cells in EBV-positive cHL can downregulate immune-dominant EBV nuclear antigens such as EBNA3A, EBNA3B and EBNA3C so that most CTLs against potential antigens lose their effect. However, the latent proteins LMP-1 and LMP-2 expressed by tumour cells in approximately 40% of patients with Hodgkin’s or non-Hodgkin’s lymphoma then become the immunotherapeutic targets of CTLs (95) (**Figure 3**). M A Roskrow et al. conducted a number of phase I clinical trials in patients with cHL using polyclonal EBV-CTLs and CTLs rich in precursors targeting LMP-2 (37). A large number of EBV-specific T cells were obtained from 9 patients with advanced Hodgkin’s disease and successfully expanded slowly *in vitro*. After these cells were injected into patients with multiple relapses, the activity of EBV-specific CTLs increased, which enhanced the immune response to EBV for more than 13 weeks. In addition, it was found that these CTLs could recognize LMP2A expressed by tumour cells (**Table 1**).

### Gene Therapy

The transcription factor EBNA2, a latent viral gene expressed by malignant cells latently infected with EBV, is a transactivator of virus and gene expression (96). M Franken et al. showed that use of the BamHI C promoter (CP) of EBNA2 in B-cell lymphoma can regulate the expression of the suicide gene and can selectively enhance the sensitivity of EBNA2-expressing cells to ganciclovir. These results provide *in vivo* and *in vitro* support for gene therapy based on the molecular basis of tumour development (96). Jian Hua Li et al. reported the feasibility of adenovirus-mediated wild-type p53 (Ad5CMV-p53) gene therapy for NPC. The adenovirus vector regulates transgene expression through the EBV replication initiation site, which can accurately transmit p53 to EBV-positive cancer cells and induce apoptosis. This approach shows effective cytotoxicity that can be enhanced by ionizing radiation (97). The team then further demonstrated that the results of EBV-targeted gene therapy could be replicated in mouse tumour models, which is expected to support the translation of this strategy into the clinic for NPC patients (98). At present, there is little research on gene therapy in EBV-LPDs, although there is much space for further exploration.

## EBV-Associated T- and NK-Cell Lymphoproliferative Disorders

### Chemotherapy

Epstein Barr virus (EBV) can cause B-cell lymphoma and is also found in some T or NK cell lymphoid tumours, such as extranodal NK/T-cell lymphoma (ENKTCL), invasive NK cell leukaemia (ANKL) and chronic active Epstein Barr virus infection (CAEBV). The WHO classification of haematopoietic and lymphoid tumours was revised in 2017 (WHO 2017), and CAEBV is defined as an T cell or NK cell tumour (99). The infected cells in CAEBV are activated and clonally proliferate with the characteristics of inflammation and malignancy. Affected patients experience fatal progression after lymphoma or HLH, so treatment needs to be started before this occurs. Chemotherapy can significantly reduce disease activity and the burden of residual EBV-infected T cells, but it is usually impossible to significantly reduce EBV-DNA load before haematopoietic cell transplantation. One of the main goals of chemotherapy is to control disease and reduce the risk of transplantation-related complications. Akihisa Sawada and others selected the combination of steroids, etoposide and cyclosporine or cytotoxic chemotherapy according to the treatment of lymphoma and then performed HSCT. The OS of 79 patients with CAEBV and related diseases reached 87% (100).

The prototype of EBV-driven lymphoma is extranodal NK/T cell lymphoma of the nasal type. Due to the high expression level of P-glycoprotein in NK lymphoma cells, chemotherapy, such as CHOP- and doxorubicin-based regimens, is largely ineffective. However, the lack of asparagine synthase makes ENKTCL sensitive to L-asparaginase. DDGP (cisplatin, dexamethasone, gemcitabine and pegaspargase) was recommended as the first-line chemotherapy for NK/T-cell lymphoma in the National Comprehensive Cancer Network (NCCN) guidelines in 2020. Other schemes based on L-asparaginase, such as SMILE (dexamethasone, methotrexate, ifosfamide, asparaginase, and etoposide), also showed good efficacy; however, this regimen is highly toxic and can even lead to death (55). DDGP, P-Gemox and AspaMetDex are less toxic alternatives to SMILE (56, 57) (**Table 2**).

EBV+ PTCL-NOS is a highly heterogeneous mature post-thymic T cell tumour. CHOP is the most commonly used first-line treatment for systemic PTCL, but this disease is usually incurable. When EBV-related LPD is pathological grade 3 and the patient has severe systemic symptoms, chemotherapy can be considered to control the disease condition but cannot improve patient prognosis (101).

### Allo-HSCT

Numerous studies at home and abroad have proven that allo-HSCT can cure EBV-T/NK-LPD. In 2000, K Kawa et al. first reported the cure of a patient with CAEBV by allogeneic bone marrow transplantation, which also eradicated EBV-infected peripheral T cells and natural killer cells (102). A series of studies have proven an obvious survival benefit for patients treated with HSCT (103, 104). However, the prognosis of patients with active disease at allo-HSCT is worse than that of patients without active disease (105). Arai et al. found that 4 of the 5 patients with active disease at the time of HSCT experienced transplantation failure or recipient cell recovery. These findings suggest the importance of disease control before HSCT. Recently, Ichiro Yonese et al. evaluated 100 patients with newly diagnosed EBV-T/NK-LPD in Japan from January 2003 to March 2016 and found that the 3-year overall survival rates of chemotherapy alone, allo-HSCT after chemotherapy and allo-HSCT alone were 0%, 65% and 82%, respectively (58). Therefore, chemotherapy has not solved the problem of disease activity before transplantation. Although allo-HSCT can cure patients, the risk of complications after transplantation is high, and it is generally reserved for use in severe cases.

### Immunotherapy

#### Monoclonal Antibodies

To date, several monoclonal antibodies against human CD38 have been successfully developed, such as daratumumab, isatuximab (SAR650984) and MOR202 (59). Wang L and colleagues demonstrated that 95% of NK/T cell lymphoma (NKTCL) cases were CD38 positive and half had high CD38 expression, which was significantly correlated with adverse results, indicating the potential role of CD38 as a therapeutic target in ENKTCL (106). A subsequent case report described an Asian woman with R/R NKTCL who achieved CR with daratumumab (107). At the 2018 American Society of Haematology (ASH) meeting, preliminary results reported a good remission rate with daratumumab for the treatment of patients with R/R NKTCL, and the ORR was 35.7% (59). The latest phase 2 study of R/R NKTCL included 32 Asian patients, and the ORR reached 25% after the application of single drug daratumumab. All patients were well tolerated, but none reached CR and the response duration was short (60). This may indicate that daratumumab alone is insufficient to treat patients with invasive characteristics, especially those with poor prognosis. Whether daretouximab can be used in combination with drugs for the treatment of NKTCL remains to be further studied. The CD30 drug–antibody conjugate brentuximab vedotin is effective in NKTCL because CD30 was reported by Feng Y et al. to be expressed in approximately 50% of 622 patients (108). To date, no clinical trials have been conducted specifically for recurrent/refractory NKTCL, but two patients were reported to achieve CR after BV treatment. Hee Kyung Kim et al. reported a case of an R/R NKTCL male patient with skin lesions who received brentuximab vedotin monotherapy and achieved long-term CR after 4 cycles. Subsequently, Li Mei Poon et al. treated ENKTCL that relapsed after chemotherapy with a combination of brentuximab vedotin with bendamustine and achieved CR (61, 62) (**Table 2**).

#### Checkpoint Inhibitors

EBV-driven latent membrane protein LMP1 is overexpressed to activate the NF-κB/MAPK and JAK/STAT signalling pathways, which leads to high PD-L1 expression. Based on the involvement of these signalling pathways, anti-PD-1 antibodies and JAK1/2/3 inhibitors have been the subject of specific drug research and applications (59) (**Figure 3**). Anti-PD-1 antibodies such as pembrolizumab and nivolumab can disrupt the interaction between PD-L1 and PD-1, thereby restoring the antitumour activity of activated T cells (109). Kwong et al. Have confirmed the efficacy of pembrolizumab in NKTCL patients (63); five of the seven patients with advanced disease achieved a CR. Immunohistochemical staining of residual lesions showed only a few CD56+EBER+ cells in infiltrating lymphocytes. This finding is consistent with the hypothesis that pembrolizumab treatment allows T cells to recognize and kill EBV-infected NK/T lymphoma cells. Xin Li et al. applied pembrolizumab in seven patients with R/R ENKL after extensive pretreatment. Two had CR and two had PR. The total response rate was 57% (64). In an independent study, three patients with R/R NKTCL also achieved a clinical response with low-dose nivolumab (65). In addition to these two popular drugs, some other PD-1/PD-L1 studies have preliminary results. In a multicenter, single arm, phase 2 clinical trial, among 28 R/R NKTCL patients, 75% achieved ORR after continuous use of sindilimab for 24 months (66). The PD-L1 inhibitor avelumab also showed monodrug activity in some patients with R/R NKTCL. In the open label phase 2 study, Seok Jin Kim et al. reported a CR of 24% (5/21) and an ORR of 38% (8/21) (67). More relevant prospective studies are still in progress (**Table 3**). PRN371 is a small molecule selective JAK3 inhibitor. Nairismägi ML and his colleagues conducted a preclinical evaluation of PRN371 and found that it significantly inhibited tumour growth in an NKTCL xenotransplantation model carrying a JAK3 activating mutation, which is consistent with the *in vitro* results (69). In addition, tofacitinib can significantly inhibit JAK3 activity *in vivo* and *in vitro*, but its clinical application in cancer treatment is limited by pan-JAK inhibitory activity. Shotaro Ando et al. found that treatment of EBV-positive T and NK cell lines with tofacitinib reduced phosphorylated STAT5 levels, inhibited proliferation, induced G1 phase arrest and reduced EBV LMP1 and EBNA1 expression (70). In addition, Erika Onozawa et al. determined that the JAK1/2 inhibitor ruxolitinib can inhibit the phosphorylation of STAT3, thereby reducing the survival rate of EBV-positive cells and cytokine production in CAEBV patients (110) (**Table 2**).

**Table 2:** Summary of available therapies in EBV-T/NK-LPD.

Therapy			Patients characteristics	Efficacy	Reference
Chemotherapy		Steroids, etoposide and cyclosporine or cytotoxic chemotherapy + HSCT	CAEBV	OS: 87%	(31)
		DDGP	NKTCL	First-line treatment.	-
		SMILE regimen	NKTCL	ORR: 79%, CR: 45%	(55)
		P-Gemox	NKTCL	ORR: 80%, CR: 51.4%	(56)
		AspaMetDex	NKTCL	ORR:77.8%	(57)
		CHOP	PTCL-NOS	Usually incurable.	–
Allo-HSCT			EBV-T/NK-LPD	It is a method to cure EBV-T/NK-LPD.	(58)
Immunotherapy	Monoclonal antibody	Daratumumab	NKTCL	ORR:35.7%	(59)
				ORR:25%	(60)
		BV	NKTCL	2 patients received CR.	(61, 62)
	Checkpoint inhibitors	Pembrolizumab	NKTCL	5 of the 7 R/R patients achieved CR.	(63)
			NKTCL	2 patients received CR 2 patients received PR.	(64)
		Nivolumab	NKTCL	3 patients with R/R NKTCL achieved clinical response.	(65)
		Sintilimab	NKTCL	ORR:68%	(66)
		Avelumab	NKTCL	CR:24%, ORR:38%	(67)
		Geptanolimab	PTCL	CR:14.6%, ORR:40.4%	(68)
		PRN371	NKTCL	It can inhibit tumor growth in NKTCL xenograft model.	(69)
		Tofacitinib	NKTCL	It can inhibit JAK3 activity *in vivo* and *in vitro*.	(70)
	CTL	LMP-CTL	NKTCL	CR was 2-6 years, and 8 survived for at least 2 years.	(71)
		LMP-CTL	NKTCL	OS: 100%, PFS:90%	(72)
Epigenetic therapy	HDAC Inhibitors	SAHA	PTCL and CTCL	It can inhibit tumor growth and metastasis in NKTCL xenograft model.	(73)
		Romidepsin	PTCL and CTCL	It can induce complete and lasting response.	(74)
		Chidamide	PTCL	It has significant single drug activity and controllable toxicity	(75)
Other approaches	Proteasome inhibitor	Bortezomib+ CHOP	NKTCL	ORR: 61.5%	(76)
		Bortezomib	CTCL and PTCL	ORR: 67%	(77)
		Bortezomib	NKTCL	ORR: 42.8%	(78)

**Table 3:** Clinical trials of therapy for EBV LPDs.

	Intervention/treatment	Phase	Tumour type	ClinicalTrials.gov Identifier
CD30 monoclonal antibody	MDX-1401	Phase 1	R/R HL	NCT00634452
	BV	Phase 2	cHL, PTCL	NCT03947255
	BV/BV + bendamostine/BV+ dacarbazine/BV+nivolumab	Phase 2	HL, PTCL	NCT01716806
	BV/BV + nivolumab	Phase 2	R/R HL, NHL	NCT01703949
	BV + nivolumab	Phase 2	R/R HL	NCT04561206
	BV	Phase 2	R/R HL	NCT01508312
	BV + chemotherapy	Phase 1 and phase 2	Stage II-IV HIV associated HL	NCT01771107
	BV + irutinib	Phase 2	R/R HL	NCT02744612
	BV + nivolumab	Phase 2	R/R HL	NCT03057795
	BV + chemotherapy	Phase 2	Stage II-IV elderly HL	NCT01476410
	BV + chemotherapy	Phase 3	Stage IIB/IIIB-IVB adolescent HL	NCT02166463
	BV + chemotherapy	Phase 1 and phase 2	R/R DLBCL	NCT03356054
	BV + lenalidomide + rituximab	Phase 3	R/R DLBCL	NCT04404283
	BV	/	R/R PTCL	NCT04213209
	BV	Phase2	PTCL	NCT03947255
CTL	Rituximab + LMP-CTL	Phase 2	child PTLD	NCT02900976
	Tabelecleucel	Phase 3	PTLD	NCT03394365
	Tabelecleucel	Phase 2	EBV related diseases	NCT04554914
PD-1 inhibitor	Nivolumab + ifosfamide, + carboplatin+ etoposide	Phase2	R/R HL	NCT03016871
	Nivolumab+radiotherapy	Phase2	cHL	NCT03480334
	Camrelizumab +/— decitabine	Phase2	HL	NCT03250962
	Camrelizumab + GEMOX	Phase2	R/R HL	NCT04239170
	Sintilimab + RCHOP	Phase2	EBV+DLBCL	NCT04181489
	Tislelizumab + zanubrutinib	Phase2	EBV+DLBCL	NCT04705129
	Tislelizumab + dexamethasone, azacytidine + pegaspargase	Phase2	NKTCL	NCT04899414
	Tislelizumab + (azacytidine + lenalidomide)/(etoposide, pegaspargase)	/	NKTCL	NCT05058755
	Tislelizumab	Phase2	NKTCL/PTCL	NCT03493451
	Avelumab	Phase2	PTCL	NCT03046953
	PD-1 blocking antibody+ chidamide + lenalidomide + gemcitabine	Phase 4	PTCL	NCT04040491
	PD-1 antibody+ HDAC inhibitor	Phase 2	PTCL	NCT04512534
	Geptanolimab (GB226)	Phase 2	PTCL	NCT03502629
	Sintilimab + chidamide+ azacidine	Phase 2	PTCL	NCT04052659
	Nivolumab + cabiralizumab	Phase 2	PTCL	NCT03927105
HDAC inhibitor	Romidepsin + pralatrexate+durvalumab +5-azacitidine	Phase 1 and phase 2	R/R PTCL	NCT03161223
	HDAC inhibitor+PD-1 antibody	Phase 2	PTCL	NCT04512534
	Romidepsin + lenalidomide	Phase 2	PTCL	NCT02232516
	Romidepsin + Ixazomib	Phase 1 and phase 2	R/R PTCL	NCT03547700
	Romidepsin + lenalidomide+ CC-486 (5-azacitidine) + dexamethasone	Phase 1	PTCL. Etc.	NCT04447027
	Azacytidine + romidepsin + belinostat + pralatrexate + gemcitabine	Phase 2	PTCL	NCT04747236

PD-1 expression can be detected in 30–60% of PTCL/NOS cases (111). In a single arm multicenter phase 2 study completed in China, all R/R PTCL patients received at least one dose of geptanolimab. Of the 89 patients with FAS, 40.4% achieved ORR, and 14.6% achieved CR. Patients with PD-L1 expression ≥ 50% benefited more from treatment (68). A large number of trials of PD-1 inhibitors alone or in combination with other drugs in PTCL are still ongoing (**Table 3**).

#### CTLs

Greater than 90% of cases of natural killer (NK)/T cell NHL, nasal type, are related to latent type II EBV. Tumour cells can express the weakly immunogenic EBV antigens LMP1, LMP2 and EBNA1. The stimulation of cytotoxic T lymphocytes (CTLs) targeting LMP1 and LMP2 showed efficacy in EBV+ NKTCL. In a clinical trial of LMP-CTLs in 52 EBV-associated lymphomas in the United States, 5 of 11 NKTCL patients received CTLs as consolidation treatment after primary radiotherapy or autologous stem cell transplantation. CR was achieved for 2–6 years, 8 patients survived for at least 2 years, and the longest period of CR was 6 years. In half of the patients, EBV levels could not be detected in plasma during CR (71). One patient with refractory NKTCL after autologous stem cell transplantation also remained in CR for 2 years after CTL infusion. Cho and colleagues studied LMP-CTL treatment in 8 patients with local disease and 2 patients with advanced NKTCL. All patients achieved CR within 4 years, with OS and PFS rates of 100% and 90%, respectively (72). However, since the 5-year survival rate of radiotherapy and chemotherapy can be as high as 90%, it is not clear whether patients with early disease truly benefit from CTL infusion. In conclusion, these trials represent significant advances in NKTCL and EBV-associated lymphoma. The role of CTL treatment in maintaining local disease after first-line treatment remains to be clarified. Longer follow-up and larger studies are needed to confirm these results. At present, there is no phase III clinical trial to guide clinical treatment.

### Epigenetic Therapy

#### Histone Deacetylase Inhibitors

Reactivation of the EBV lytic cycle can enable cytotoxic antiviral drugs to achieve a specific killing effect on EBV-positive cells. These types of therapy include chemical lytic inducers and nucleoside analogue antiviral prodrugs. Studies have shown that HDAC inhibitors can reactivate the lytic cycle and lead to enhanced apoptosis of NPC and gastric cancer cells (112–114). HDAC inhibitors are divided into three categories according to their chemical structure: hydroxamate, cyclic peptide and benzamide. Suberoylanilide hydroxamic acid (SAHA) induces apoptosis and/or cell cycle arrest in some T and NK cell lines. SAHA also inhibited tumour progression and metastasis in a mouse xenograft model, demonstrating that it can inhibit EBV-related T and NK cell lymphoma (73). The cyclic peptide romidepsin has been studied by Bertrand Coiffier et al., who been found that romidepsin monotherapy can induce a complete and lasting response in patients with recurrent or refractory PTCL of all major subtypes, regardless of the number or type of previous treatments, and has controllable toxicity (74). At present, SAHA and romidepsin have been approved by the FDA for the treatment of many types of malignant tumours, such as peripheral and cutaneous T-cell lymphomas (115). Both drugs can induce an EBV lysis cycle at acceptable concentrations in patient plasma (113, 116). Chidamide is a novel oral benzamide HDAC inhibitor. In a phase II study, Y. Shi et al. evaluated the efficacy and safety of chidamide in recurrent or refractory peripheral T-cell lymphoma (PTCL) in a Chinese population and found that it has significant single-agent activity and controllable toxicity (75). Seventy-nine patients were enrolled, and an ORR of 28% was reported; 14% of the patients achieved a CR/CRu, and the OS was 21.4 months. Among these patients, the effects were more pronounced and the response was more durable in AITL patients. Most adverse events were limited to grades 1–2. Based on the results of this critical trial, CFDA approved chidamide for this indication in December 2014. However, it should be noted that HDAC, especially romidepsin, may lead to serious adverse events based on the mechanism of EBV cleavage recirculation activation. In an open label prospective pilot study, SJ Kim et al. found that three of the five NKTCL patients treated with romidepsin had a rapid increase in EBV DNA titer in their blood and an increase in liver enzymes and bilirubin levels (117). Therefore, patients with NKTCL should avoid using romidepsin, but patients with recurrent refractory diseases without other treatment options can consider the combination of two or more research drugs with strong biological principles. Joo Hyun Kim et al. conducted high-throughput screening of FDA approved drugs and tested them in EBV positive cell lines. It was found that phosphodiesterase 5 (PDE5) inhibitors, such as sildenafil, seem to be non-toxic and effective inhibitors of romidepsin induced EBV reactivation (118). A large number of clinical trials of HDAC inhibitors combined with other drugs are ongoing (**Table 3**).

### Other Approaches

#### Proteasome Inhibitors

Proteasome inhibitors have been shown to inhibit cell growth and promote cell death in a variety of cancers. Bortezomib is the first proteasome inhibitor approved by the FDA in 2003 for the treatment of multiple myeloma (MM) and mantle cell lymphoma (119). The manipulation of normal ubiquitin proteasome system function by EBV is vital for virus replication and the survival of virus-infected cells. EBNA-1, LMP-2A and LMP-1 inhibit proteasome-mediated degradation to maintain virus latency, while bortezomib can reactivate the EBV lytic cycle in EBV-related BL cells (120). Induction of the EBV lytic cycle can activate the radioisotope [^125^I]2’-fluoro-2’-deoxy-β-D-5-iodouracil-arabinofuranoside, selectively inhibiting the growth of BL xenografts in severe combined immunodeficiency (SCID) mice (121). Granato M et al. found that bortezomib activates endoplasmic reticulum (ER) stress and that C/EBP-β, C-Jun N-terminal kinase (JNK) and autophagy mediate the transformation of a latent to a lytic EBV infection. It is more difficult to induce the EBV lysis cycle in lymphoid cells than in epithelial cells, and this effect is limited to BL cells (122). In addition to reinducing the lysis cycle, bortezomib can affect the apoptosis of EBV-related malignant tumour cells. The latent EBV membrane protein LMP-1 has been shown to activate the NF-κB pathway in BL and NPC, and this pathway has strong tumorigenic activity and may contribute to resistance to apoptosis inducers (123, 124). Bortezomib can protect the inhibitory protein IκBα and block NF-κB activation, leading to malignant cell apoptosis (125–128). It has been reported that bortezomib can induce the apoptosis of NK lymphoma/leukaemia cells, and this result was verified *in vivo* (76, 77, 129). In a phase I study, bortezomib combined with CHOP was administered to patients with advanced invasive T-cell lymphoma, and the ORR was 61.5%. Pier Luigi Zinzani et al. obtained a 67% ORR in patients with cutaneous T-cell lymphoma and invasive T/NK cell lymphoma treated with bortezomib monotherapy. In a study involving 6 NKTCL patients, the ORR after treatment with the B-GIFOX (bortezomib, gemcitabine, oxaliplatin and ifosfamide) regimen was 42.8% (78).

#### Selective Nuclear Export Protein Inhibitors

XPO1 is a nuclear export protein responsible for transporting biological macromolecules from the nucleus, including tumour suppressor proteins (TSPs) (e.g. p53, p73, FOXO3a, and IκB), growth regulators (e.g., glucocorticoid receptors), and oncogenic proteins (e.g., mRNA, c-myc, cyclin D, Bcl-2, and Bcl-6). In healthy cells, this process is strictly regulated to maintain an appropriate balance between cell growth and apoptosis (130). Studies have found that high levels of XPO1 are associated with poor clinical prognosis of multiple myeloma, DLBCL, glioblastoma and other diseases (130–132). As a linker protein, the EBV protein SM is involved in the nuclear output of mRNA encoding the soluble EBV gene. XPO1 inhibitors can covalently bind to XPO1 active sites and inhibit the output of corresponding mRNAs to the cytoplasm for translation, thereby effectively inhibiting EBV replication and virus transmission (133). Based on the mechanism of action of XPO1 inhibitors, they have broad application prospects in EBV-LPD and are under clinical investigation. A double-blind, placebo-controlled, phase I study of ATG-527 (KPT-335, verdinexor) for the treatment of CAEBV is ongoing; the aim of this study is to evaluate the safety, tolerability, pharmacokinetics and overall treatment response of different dose levels of ATG-527 in patients with CAEBV.

#### Exosomes

Exosomes are small membrane-bound vesicles secreted by cells. These extracellular vesicles (EVs) carry a wide range of molecules and affect intercellular communication, which contributes to the pathogenesis of various diseases and infections (134, 135). There is considerable evidence that EBV-related exosomes specifically package a variety of viral components that may promote EBV infection (such as LMP-1, lmp-2a, EBER, viral RNA, and miRNA) (136–139), which may help EBV establish the surrounding tumour microenvironment to promote tumour growth and survival. In addition, Keryer-Bibens C et al. found that galectin-9-containing exosomes inhibited EBV-specific T cell proliferation and induced apoptosis (136). Therefore, blocking these exosomes can restore immune surveillance while resisting tumours. Exosomes have been designated as an effective target for cancer treatment, but they need to be further explored.

## Conclusion

In general, there has been great progress in our understanding and treatment of EBV-LPDs in recent years from traditional chemotherapy, changes in immunosuppressants and HSCT to immunotherapy, gene therapy and epigenetic therapy stemming from discoveries of signaling pathways and virus latency and lysis cycles; together, this improved understanding will lead to the development of better treatment options with fewer side effects. Among such potential treatments, proteasome inhibitors, HDAC inhibitors and JAK inhibitors have been tested *in vitro* and *in vivo* using xenotransplantation models. The effects of rituximab, nivolumab, pembrolizumab, daratumumab, bortezomib and CTLs have been confirmed in clinical trials. However, we must appreciate that most of the research results are limited to specific groups. For heterogeneous populations of EBV-LPDs, immunotherapy targets, checkpoint inhibitors, CTLs, small molecule targets and even gene therapy are still elusive. We hope that as the data and science related to these methods mature, they will provide alternative treatments as both monotherapy and combination therapy.

## Author Contributions

KL and TY design, analysis and draft the manuscript. MY, ZC: supervision, funding acquisition, writing–review and editing. YZ: methodology, and writing–review and editing. FL: design, supervision, funding acquisition, methodology, project administration, writing–review and editing. All authors contributed to the article and approved the submitted version.

## Funding

This work was financially supported by grants from the National Natural Science Foundation of China (81960043, 82160043), Natural Science Foundation of Jiangxi Province (20192ACB20030), Science and Technology Innovation Base Construction Project of Jiangxi Province (20211ZDG02006, 20212BCG74001).

## Conflict of Interest

The authors declare that the research was conducted in the absence of any commercial or financial relationships that could be construed as a potential conflict of interest.

## Publisher’s Note

All claims expressed in this article are solely those of the authors and do not necessarily represent those of their affiliated organizations, or those of the publisher, the editors and the reviewers. Any product that may be evaluated in this article, or claim that may be made by its manufacturer, is not guaranteed or endorsed by the publisher.
